# Prescribing trends of antipsychotics in youth receiving income assistance: results from a retrospective population database study

**DOI:** 10.1186/1471-244X-13-198

**Published:** 2013-07-27

**Authors:** Andrea L Murphy, David M Gardner, Charmaine Cooke, Steve Kisely, Jean Hughes, Stan P Kutcher

**Affiliations:** 1 College of Pharmacy and Department of Psychiatry, Dalhousie University, 5968 College St, PO Box 15000, Halifax, NS, B3H 4R2, Canada; 2 Department of Psychiatry and College of Pharmacy, Dalhousie University, QEII HSC, AJLB 7517, 5909 Veterans' Memorial Lane, Halifax, NS, B3H 2E2, Canada; 3 Department of Health and Wellness, Joseph Howe Building, 1690 Hollis Street, PO Box 488, Halifax, NS, B3J 2R8, Canada; 4 University of Queensland, School of Population Health, Herston, QLD, 4006, Australia; 5 School of Nursing, Dalhousie University, 5869 University Avenue, PO Box 15000, Halifax, NS, B3H 4R2, Canada; 6 Sun Life Financial Chair in Adolescent Mental Health, Dalhousie University/IWK Health Centre, 5850 University Ave, PO Box 9700, Halifax, NS, B3K 6R8, Canada

**Keywords:** Antipsychotics, Pediatrics, Retrospective studies, Pharmacoepidemiology

## Abstract

**Background:**

Prescribing of antipsychotics (AP) to young people has increased in the last decade internationally. We aimed to characterize AP prescribing in a population of low-income youth in Nova Scotia, Canada.

**Methods:**

We conducted a population database study of AP prescription claims and health services utilization by young people aged 25 years and younger receiving drug benefits through the publicly funded Pharmacare program between October 1, 2000 to September 30, 2007.

**Results:**

Four percent (1715/43888) of youth receiving Pharmacare benefits filled AP prescriptions. The use of second generation antipsychotics (SGAs) significantly increased (p < 0.0001) in all age groups except 0-5 year olds, whereas first generation antipsychotic use significantly decreased. Pharmacare beneficiaries aged 21-25 years represented 45.2% of AP users. The majority (66%) of youth filling AP prescriptions had 2 or more psychiatric diagnoses. Most youth (76%) filled prescriptions for only one type of AP during the study period. Psychotic disorders were the most common indication for AP use except with risperidone, in which ADHD was the most likely reason for use. Co-prescribing of psychotropics was prevalent with antidepressants and mood stabilizers prescribed in 42% and 27% of AP users, respectively. General practitioners (GPs) prescribed incident APs most often (72%) followed by psychiatrists (16%). The age- and gender-adjusted rate of death was higher in AP users as compared to the age-matched general population of Nova Scotia.

**Conclusions:**

SGA use increased significantly over seven years in a cohort of 0 to 25 years olds receiving Pharmacare benefits. Off-label use of APs was prevalent with ADHD and other non-psychotic disorders being common reasons for AP use. GPs initiated most AP prescriptions. Co-prescribing of other psychotropics, especially antidepressants and mood stabilizers, was prevalent even in younger age strata. This study raises further questions about AP prescribing in those 25 years of age and under, especially given the range of diagnoses and psychotropic co-prescribing.

## Background

The use of antipsychotic (AP) and other psychotropic medications in children and adolescents is a complex phenomenon with many aspects yet to be satisfactorily characterized. Several studies have demonstrated a dramatic rise in the use of second generation antipsychotics (SGAs) in young people, which has intensified concerns regarding their overall safety and effectiveness profile in this age group [1-22].

We suspected from our clinical and literature-based knowledge that AP use in youth, particularly in those with lower incomes, was increasing in Nova Scotia. We conducted a mixed methods study with an initial quantitative phase followed by a qualitative phase. Using a retrospective population database design, we aimed to characterize AP prescribing by prevalence, drug, dose, duration, indication/diagnoses, prescriber type, psychotropic co-prescribing, morbidity, and mortality in a population of low income youth.

## Methods

### Patients - cohort and databases

This study was conducted in Nova Scotia, which is a Canadian province of approximately 940,000 residents. The cohort in this retrospective population based study of prescription drug claims from October 1st, 2000 (2000Q3) to September 30, 2007 (2007Q2) included all Pharmacare beneficiaries 25 years of age or younger receiving Community Services in Nova Scotia. The prescription claims are housed at the Population Health Research Unit (PHRU) at Dalhousie University and specific procedures and criteria are required for acquisition of data [23]. By agreement with the Nova Scotia Department of Health PHRU holds anonymous, encrypted data files concerning demographics on prescribers and drug benefit data. The province’s Department of Community Services provides prescription drug coverage (i.e., Pharmacies) to income assistance clients, Services for Persons with Disabilities clients, children in the care of child welfare through either a district office of the Department of Community Services or a Children's Aid Society/Family and Children's Services agency and low income Pharmacies for Children clients. Eligibility for the program can change depending on personal circumstance (e.g., change in employment, loss fixed address) and as such, beneficiaries can enter and leave the database depending on meeting the eligibility requirements. Periods and dates of interruption in benefits can be tracked in the databases. As such, a definition for “long-term user”, which includes individuals with longer exposure to APs, was defined as individuals with two or more prescriptions for APs within 180 days and the cumulative days supply from these prescriptions was required to be a minimum of 90 days. Analyses were conducted on this group to determine rates of co-prescribed psychotropics and non-psychiatric comorbidity.

PHRU also houses data from other population databases that capture health services utilization information including Vital Statistics (causes of death), the Canadian Institute for Health Information Discharge Abstract Database (CIHI DAD; demographic and diagnostic information for hospitalizations in Canada), which includes demographic and diagnostic information of all hospitalizations in Canada, and the Nova Scotia Medical Services Insurance (MSI) Physician Billings (all fee-for–service claims and shadow-billings by physicians and nurse practitioners including data of service, specialty, diagnosis and patient demographics). These databases were linked to the encrypted unique identifiers of AP prescription recipients to determine health services utilization including hospital admissions, diagnoses, visits, and prescriber types. PHRU also has a file to determine patient residence that is based on postal code. Specific details of the databases used are available through PHRU [23].

### Identification of antipsychotic use

First generation antipsychotic (FGA) and second generation antipsychotic (SGA) prescriptions were identified using the WHO Anatomical Therapeutic Classification (ATC) code to the 7^th^ level (Table 1) for agents available in Canada during the study period [24]. Market availability of some APs (e.g., thioridazine) changed during the study period and others (e.g., SGAs) were subject to specific criteria for reimbursement by the Pharmacare program (Table 1), which changed over time. Where several formulations were available (e.g. long-acting injection and oral) drug identification numbers (DINs) were used to differentiate the products. Short acting intramuscular use was not targeted given that these formulations are typically reserved for hospital or inpatient use. For most results FGAs were grouped given their relatively infrequent use compared to SGAs.

**Table 1:** Antipsychotic WHO Anatomical Therapeutic Codes

**ATC code**	**Drug name**	**Formulations in Canada**
N05AA01	chlorpromazine	Oral
N05AA02	methotrimeprazine	Oral
N05AB02	fluphenazine	Oral & long-acting injection
N05AB03	perphenazine	Oral
N05AB06	trifluoperazine	Oral
N05AC01	peryciazine	Oral
N05AC02	thioridazine*	Oral
N05AC04	pipotiazine	Depot
N05AD01	haloperidol	Oral & long-acting injection
N05AF01	flupenthixol	Oral & long-acting injection
N05AF04	thiothixene	Oral
N05AF05	zuclopenthixol	Oral & long-acting injection
N05AG02	pimozide	Oral
N05AH01	loxapine	Oral
N05AH03	olanzapine†	Oral
N05AH04	quetiapine‡	Oral
N05AX08	risperidone‡	Oral & long-acting injection

* Removed from Canadian market 2005.

† Specific criteria (e.g. initiated by psychiatrist) for use must be demonstrated before Pharmacare coverage is provided.

‡ Specific criteria for Pharmacare coverage were in place until December 2003 after which it became a drug benefit without restrictions on use.

Incident prescription of an AP was defined as the first recorded AP prescription during the observation period of October 1^st^, 2000 to September 30, 2007. It excluded those individuals with AP prescriptions in the 6 months prior to October 1, 2000. In each quarter, a prevalent user was an individual for whom an AP prescription was dispensed. The Pharmacare program reimburses a maximum of 100 days of dispensed medication at each prescription fill.

### Identification of co-prescribed psychotropics

Co-prescribed psychotropic medications were defined as prescription claims occurring within 90 days prior to the incident AP prescription and up to 180 days after the date of the incident AP prescription in long-term antipsychotic users. Medications of interest included antidepressants, mood stabilizer, anxiolytics, and ADHD related medications (e.g., stimulants, atomoxetine, and clonidine). Medications were targeted using 7^th^ level ATC codes (the complete list of medications and ATC codes are available on request).

### Identification of diagnoses

**Figure 1:**
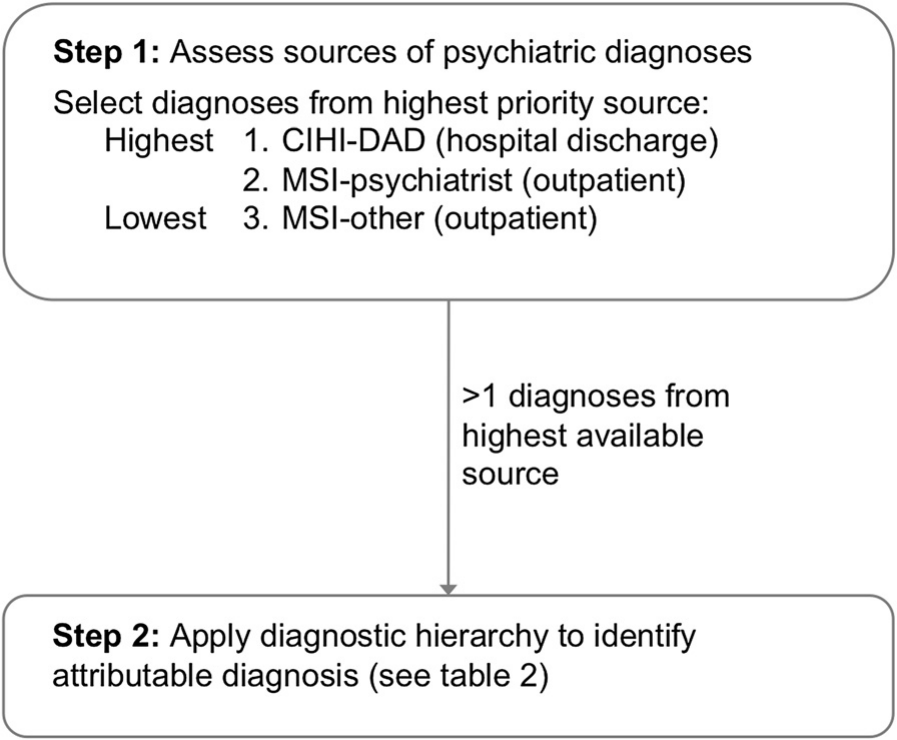
Stepwise procedure for identifying the most attributable diagnosis for antipsychotic users with more than one diagnosis*. *66% of antipsychotic users had more than one psychiatric diagnosis three months before or after the incident antipsychotic prescription.

International Classification of Diseases (ICD) 9 and 10 codes were used for diagnostic information from the MSI Physician Billings database and CIHI DAD, respectively. As diagnostic information is not included on prescription drug claims data for Pharmacare, diagnoses most attributable to AP use were established according to a timeline and hierarchy in relation to the AP prescription. The procedure for establishing the diagnoses was developed by the research team based on knowledge of literature [4,25], clinical experience, and properties of the included databases. This approach to establishing diagnoses is not dissimilar from other researchers utilizing claims data without available diagnosis per prescription [4,25]. For our study, a diagnosis was eligible to be attributable to AP use if it had occurred three months before or after the incident AP prescription. A diagnostic hierarchy was applied to cases in which more than one diagnosis occurred during this time period, a hierarchy to determine the most attributable diagnosis for AP use was employed. The hierarchy included which database the diagnosis came from, the provider type (e.g., psychiatrist vs. general practitioner), and the diagnoses itself (e.g., psychosis vs. conduct disorder) (Figure 1). For databases, CIHI-DAD diagnoses received priority over MSI diagnoses. For example, if two diagnoses were received around the time of the incident AP prescription and one diagnosis was from a mental health related hospitalization (i.e., CIHI DAD) and another from a general practitioner per the MSI database, the hospitalization diagnosis was designated as the most attributable to AP use. If MSI only diagnoses were available, diagnoses assigned by psychiatrists were given priority over other providers (e.g., general practitioners). Also, if one or more diagnoses were received and could not be differentiated based on database source or provider type, the next step was to prioritize the diagnoses on the hierarchy outlined in Table 2. As an example, if a person received several diagnoses during a hospitalization (e.g., psychosis, tics, and alcohol abuse), which would be found in the CIHI-DAD database, the most attributable diagnosis for AP use according to the hierarchy is psychosis (Table 2). ICD-9 and 10 codes for groupings of diagnoses contained in the hierarchy are given in Table 2.

**Table 2:** Attributable diagnostic hierarchy for type of diagnosis when more than one diagnosis occurred temporally with AP use

**Level**	**Category**	**ICD-9 codes**	**ICD-10 codes**
1	Psychotic disorders (non-affective), organic psychoses	291.3, 295 and 297, 298, 293.0, 293.1, 293.81, 293.82, 293.83	F20-29, F06, F09
2	Bipolar	296.0, 296.1, 296.4, 296.5, 296.6, 296.7, 296.8, 296.9	F30, F31, F34.0, F38.0
3	Pervasive Developmental Disorders/Mental Retardation	299, 317-319	F70-F79, F84, F89
4	Tics	307.2	F95
5	Other disruptive disorders (oppositional defiant disorder, conduct disorder, impulse control disorders, personality disorders)	301, 312, 313	F60 - F62, F63, F68, F91, F92
6	Attention deficit hyperactivity disorder	314	F90, F98.8
7	Depression (major depressive disorder)	296.2X* - 296.3X*, 300.4, 311	F32, F33, F34.1, F38.1, F39
8	Anxiety disorders (neuroses), adjustment disorders, sleep disorders	300, 307.4, 308, 309, 310, 780.5	F40-F42, F43, F44-F48, F51, F93.1-93.9, F07
9	Eating disorders	307.1, 307.51, 307.5	F50
10	Other (sexual disorders, alcohol & substance abuse/dependence, learning disabilities)	302, 303-305, 315	F10-F19, F65, F66, F06, F07, F80, F81, F82, F83

* “X” included in the code instructed the data analyst to include any number that followed the preceding digit.

Non-psychiatric comorbid diagnoses, including those more commonly associated with APs, were targeted using ICD 9 and 10 codes in the MSI and CIHI databases for long-term users of APs. Diagnoses such as cardiovascular disorders, endocrine disorders, and movement disorders were of interest. They were eligible for consideration if they occurred within two years after the first prescription for the incident long-term AP prescription, and not in the 12 months previous to the prescription.

### Identification of dose and duration of use

Mean prescribed daily doses were calculated for each AP using the days supplied field. Duration of use for unique identifiers was calculated for those who were in the cohort for long periods (i.e., ≥365 days).

### Identification of prescriber from drug claims

Provider specialty was identified from a specific field in the drug claims data for incident AP prescriptions. Categories of interest included: general practice, pediatrics, psychiatry, emergency medicine, internal medicine, neurology and pediatric neurology. Nurse practitioners did not have prescriptive authority for APs in the community setting during the study and therefore were not included as prescribers.

### Identification of mortality rates

Cases of death were obtained by linking with vital statistics data.

### Costs

Annual costs were calculated based on amounts paid by Pharmacare to the pharmacy minus the fee paid by AP users.

### Statistical analyses

Descriptive statistics including mean, median, standard deviations and interquartile ranges (IQR) were used for several aspects of AP use. Stratifications by age, gender and location were used for several analyses.

Time series analyses were used to identify for trends in incident and prevalent AP use over the duration of the observation period. The correlogram for each age group indicated that the data are not stationary because there were many autocorrelation values outside the 2 standard error band. After taking the difference once, the correlogram showed that the correlation values died down quickly indicating that the model should have an autoregressive component of the order 1. The time series regression model was Y(t) = incidence or prevalence rate at time t (quarter), which is Y(t) = A + B*t + a(t) where a(t) + k*a(t-1) = e(t). Here t-1 is the 1 quarter before time “t”, A is the intercept, and B is the regression parameter. It was assumed that the error term a(t) can be AR(1) model, that is, a(t) = e(t)-k*a(t-1) where k is the autocorrelation coefficient. The null hypothesis was that no change in incidence and prevalence occurred over time and the alternative was that the rates did change over time. The p value for significance was 0.05. Time series were conducted for age, gender, and location for quarterly incidence and prevalence We conducted a Cox proportional hazards regression to model survival, controlling for age and gender. Kaplan-Meier survival curves were generated. ANOVA was used to compare means and Chi square was used for intergroup comparison of dichotomous variables.

### Ethical considerations

This study received ethical approvals from the IWK Health Centre and the Capital District Health Authority. An approval from PHRU’s Data Access Committee outlining adherence to criteria in the Data Access Guidelines [23] is also required prior to receiving permission to access data housed at PHRU.

## Results

Approximately four percent (1715/43888) of those aged 25 years old or less who were eligible to receive Community Services benefits received AP prescriptions during the study period of October 1, 2000 to September 30, 2007 (2000Q3 to 2007Q2) (Table 3). Over a third (519/1715) of all AP users were 15 years of age or younger and over half (55%) of AP usage was attributable to beneficiaries 20 years of age and less. Of those Community Services beneficiaries who were 20 years and less, nearly 3 percent received prescriptions for APs (939/35,801) (Table 3). Overall, the odds of receiving an AP was 2.5 times greater in males compared to females (95% CI 2.3, 2.8; X^2^ = 338 df = 1, p = <0.0001). Time series analyses for overall prevalence of AP use by gender was significant over time for males (p < 0.0001). Seventy six percent (N = 1306) of our sample received one AP with a smaller proportion receiving two (N = 287), three (N = 81) or, four or more (N = 41). A substantial portion of AP users met the criteria of a long-term user (N = 1270).

**Table 3:** Antipsychotic use by age and gender in Community Services Pharmacare beneficiaries from October 1, 2000 to September 30, 2007

**Age group**	**Total Community Services Pharmacare beneficiaries (% male)**	**Antipsychotic users of Community Services Pharmacare beneficiaries by age (%)**	**Male antipsychotic users of male Community Services Pharmacare beneficiaries by age (%)**	**Female antipsychotic users of female Community Services Pharmacare beneficiaries by age (%)**
0-5	14800 (51.6)	43 (0.3)	35 (0.5)	8 (0.1)
6-10	7474 (51.8)	207 (2.8)	170 (4.4)	37 (1.0)
11-15	6693 (47.6)	269 (4)	192 (6.0)	77 (2.2)
16-20	6834 (35.4)	420 (6.1)	269 (11.1)	151 (3.4)
21-25	8087 (29.9)	776 (9.6)	468 (19.3)	308 (5.4)
Total 0-25	43888 (44.5)	1715 (3.9)	1134 (5.8)	581 (2.4)

**Figure 2:**
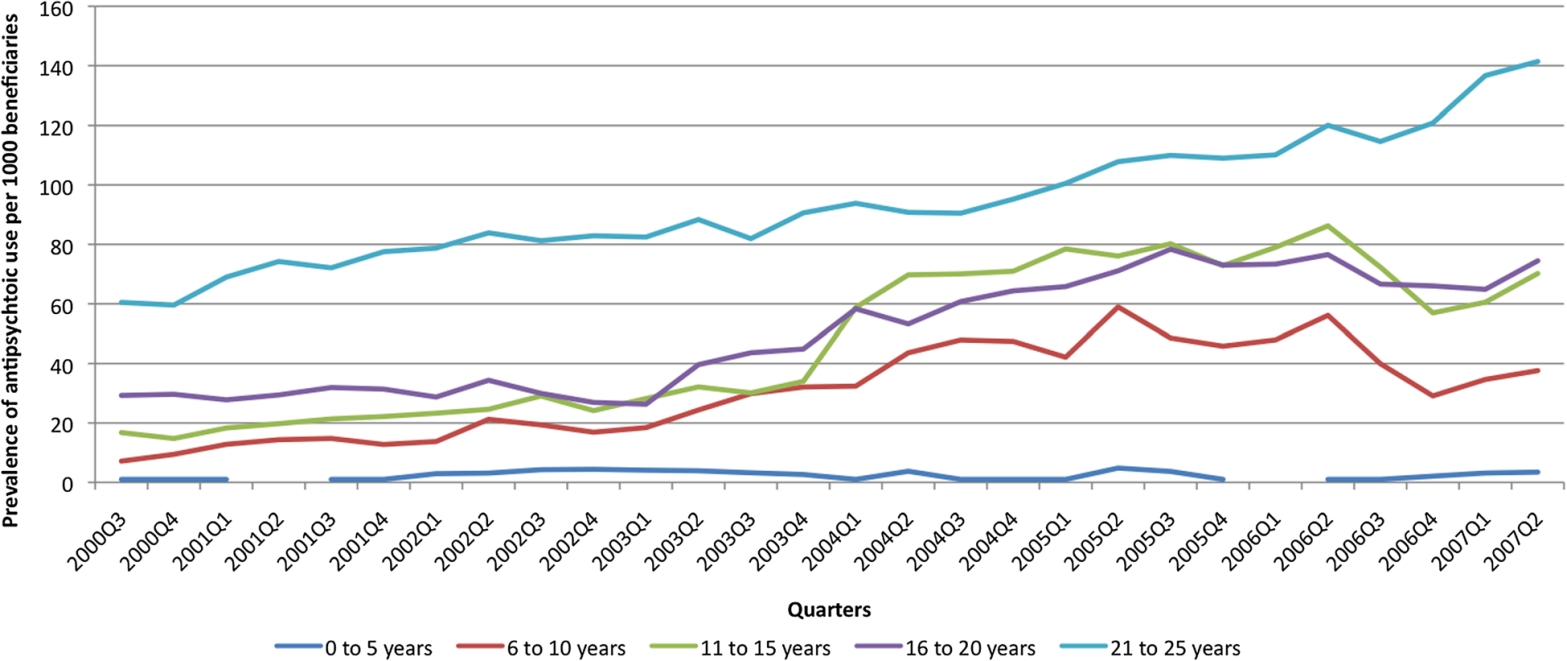
Quarterly prevalence of antipsychotic use by age group* per 1000 beneficiaries, October 1, 2000 (2000Q3) to September 30, 2007 (2007Q2). * In the 0 to 5 age group, the line is not continuous due to cell counts that were zero.

Over time, the prevalence of AP use increased (p < 0.0001) (Figure 2) except in 0 to 5 year olds.

**Figure 3:**
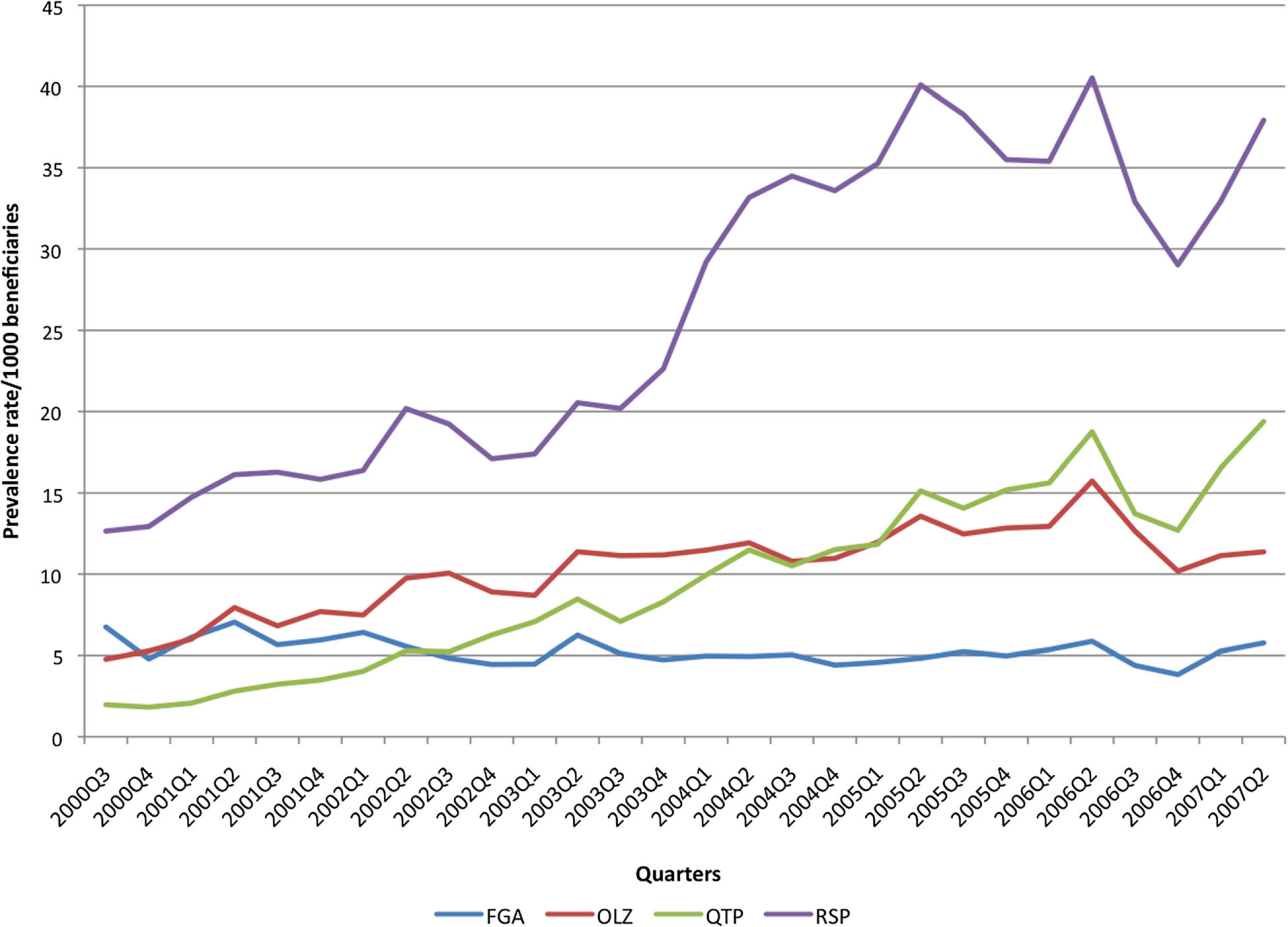
**Use of first and second generation antipsychotics per 1000 beneficiaries October 1, 2000 (2000Q3) to September 30, 2007 (2007Q2).** FGA: first generation antipsychotics; OLZ: olanzapine; QTP: quetiapine; RSP: risperidone.

SGA use rose significantly as a group and by individual agent, whereas FGA use decreased significantly (p < 0.005) (Figure 3). Risperidone use was the most commonly used AP. Long-acting injectable use over the study period was 3.3% (57/1715) and varied per year such as 1.5% in 2005 and 4.6% in 2002.

### Prescribers and diagnoses

Prescriber type was available for 52% of incident antipsychotic prescriptions. From the available data, general practitioners (GPs) initiated the majority of these AP prescriptions (72%, 646/898), with psychiatrists initiating 16% (144/898) and pediatricians 3.5% (31/898). Other providers (e.g., emergency medicine, neurology) accounted for a small proportion of prescribing (approximately 2%).

Thirty-four percent had one psychiatric diagnosis and for those with more than one diagnosis (66%) we applied our hierarchy. Hospital discharge information was used as the source of diagnosis in 36% of AP users. The remainder of the diagnoses were made in ambulatory care settings by psychiatrists (16%) and other physicians (48%). The diagnostic source varied markedly by the type of diagnosis. Hospital discharge diagnoses were the most frequent source when the diagnoses were psychoses (66%), other disruptive disorders (61%), and bipolar disorder (49%). General practitioners and other non-psychiatrist physicians were the most frequent diagnostic source for other DSM-IV diagnoses (87%, 102/117), anxiety disorders (79%, 207/263), ADHD (75%, 205/273), depression (52%, 70/135), and pervasive developmental disorder/mental retardation (52%, 37/71).

Psychosis (31%) was the most common attributable diagnosis when an antipsychotic was initiated, followed by ADHD (16%), anxiety disorders (15%), other disruptive disorders (10%), depression (8%), bipolar disorder (7%), other miscellaneous DSM-IV diagnoses (7%), pervasive developmental disorder/mental retardation (4%), tics (2%), and eating disorders (<1%). Sixty-six percent (940/1419) of AP prescription recipients had two or more psychiatric diagnoses in the 3 months prior to AP prescription and up to the date of the last fill for that AP. No AP related diagnostic information could be determined for 296 AP prescriptions during this time. Neuroses (anxiety and somatoform disorders) was the most common diagnosis by count and was recorded for 573 recipients.

**Figure 4:**
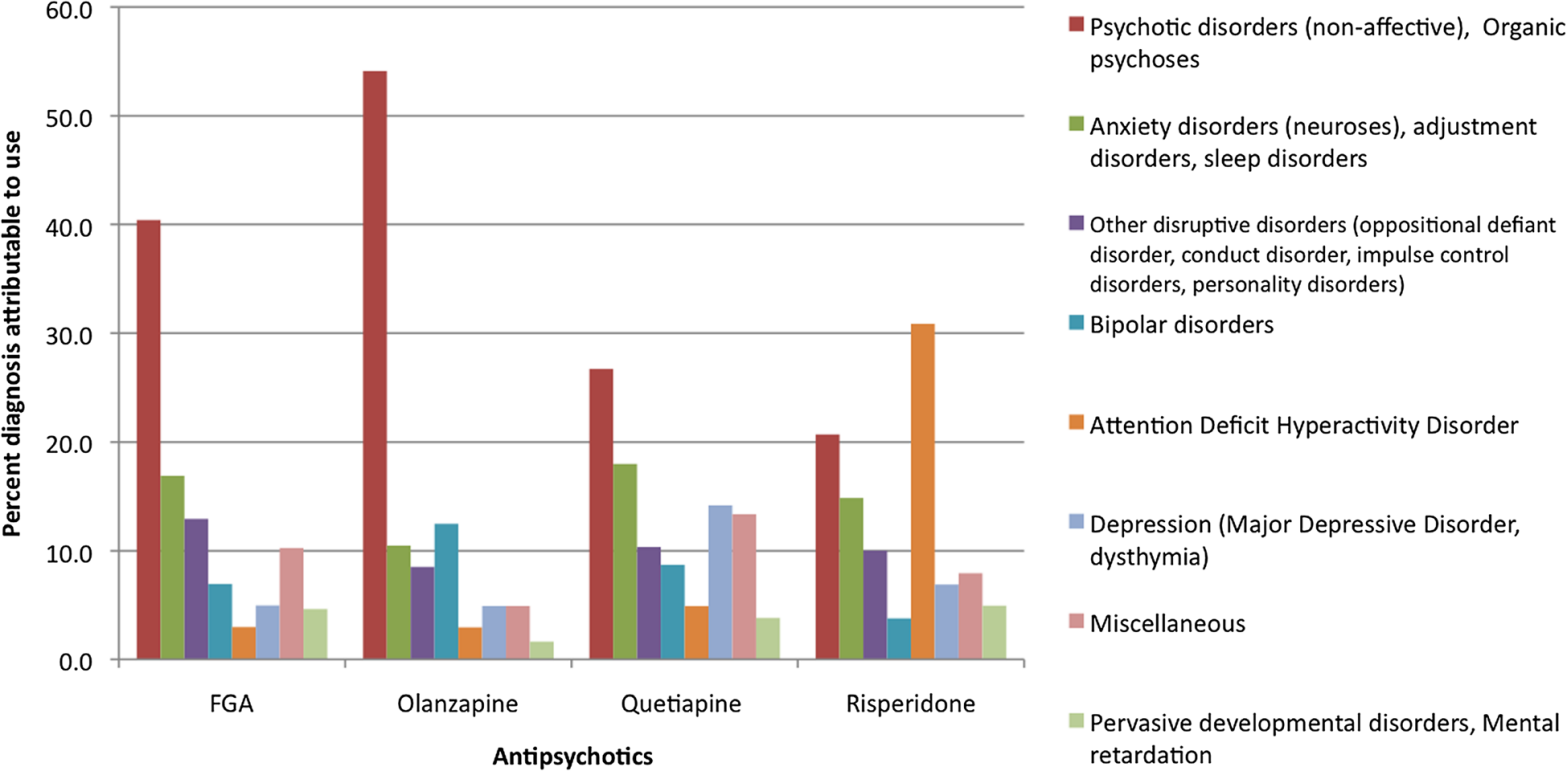
Percentages of attributable diagnoses to antipsychotic use (N = 1419).

Psychotic disorders were the most common attributable reason for AP use for FGAs (40%), olanzapine (54%), and quetiapine (27%) in those with available diagnoses (N = 1419) within 3 months before the incident prescription and up to the last prescription for the AP. Attention Deficit Hyperactivity Disorder (ADHD) was the most attributable diagnosis for risperidone use (31%) followed by psychosis (21%). Combined anxiety disorders (neuroses), adjustment disorders, and sleep disorders attributed to 17% of FGA use, 11% of olanzapine use, 18% of quetiapine use, and 15% for risperidone. For graphical representation purposes, eating disorders, tics, and other indications (i.e. sexual disorders, alcohol and drug abuse related disorders, and learning disabilities) were combined into one category called miscellaneous (Figure 4).

### Doses prescribed

**Figure 5:**
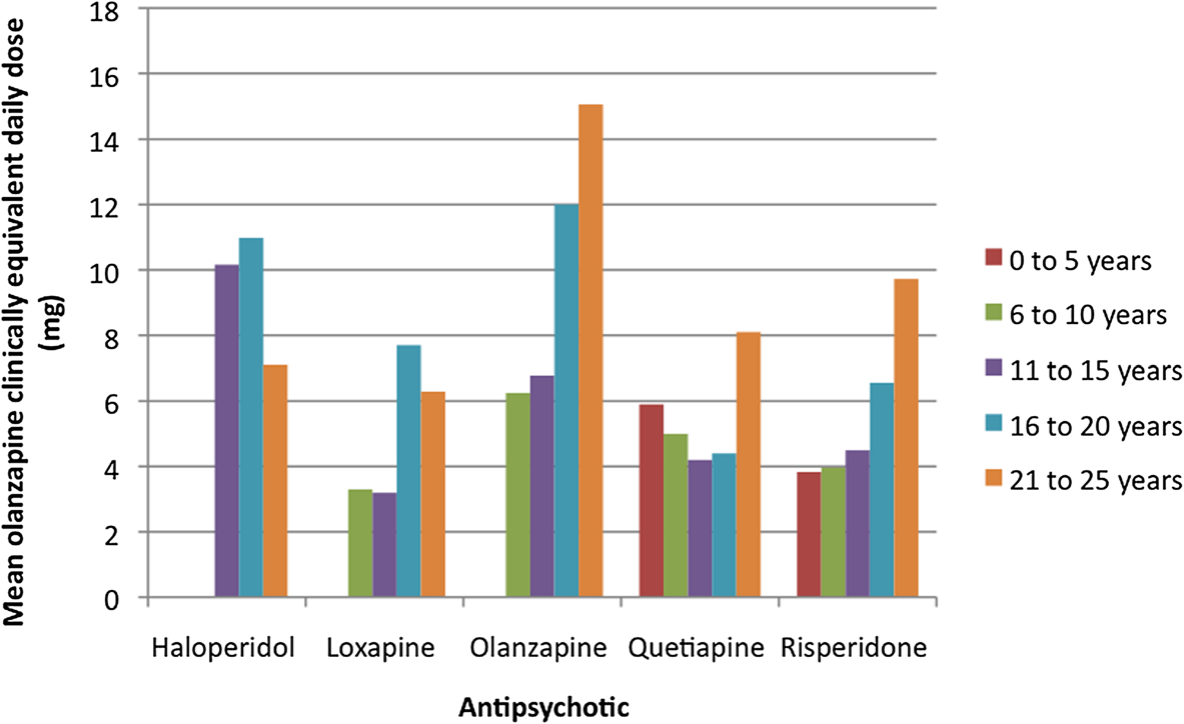
Age* stratified mean olanzapine clinically equivalent† antipsychotic daily doses in 2006. *No haloperidol prescriptions were evident for 0 to 10 year olds and no 0 to 5 year olds had prescriptions for loxapine or olanzapine. †Clinically equivalent doses compared to olanzapine were calculated using equivalency ratios of 2 for haloperidol, 0.33 for loxapine, 0.027 for quetiapine, and 3.33 for risperidone [26].

The annual mean dose per AP user, stratified by age, for 2002 and 2006 is reported in Table 4. Average dose per age group was stable or slightly decreased over this time period. Figure 5 shows the mean overall olanzapine-equivalent dose [26] per age group for each antipsychotic. Dosing of olanzapine was higher than dosing of the other SGAs.

**Table 4:** Mean (SD) doses of antipsychotics

	**Quetiapine**		**Risperidone**		**Olanzapine**		**Haloperidol**		**Loxapine**	
Year	2002*	2006	2001	2006	2001	2006	2001	2006	2001	2006
Mean mg dose (SD)	306 (258)	266 (405)	2.9 (2.4)	2 (3.3)	13 (7.2)	13.6 (9.6)	5 (4.1)	4.6 (4)	32.3 (40.7)	16.8 (22)
N†	567	1375	1432	2547	633	1424	130	78	50	229

* 2002 was chosen due to quetiapine’s release date on the market.

†N represents the number of prescriptions for that antipsychotic during the year.

### Duration

The median (IQR) duration of AP use for people who were in the Community Services cohort for at least 365 days (N = 1528) was 290 days (90, 808), 270 (90, 660), 369 (135, 930), 299 (100, 763), 332 (72, 1035), 300 (90 to 861) for those aged 0 to 5, 6 to 10, 11 to 15, 16 to 20, and 21 to 25, respectively.

**Figure 6:**
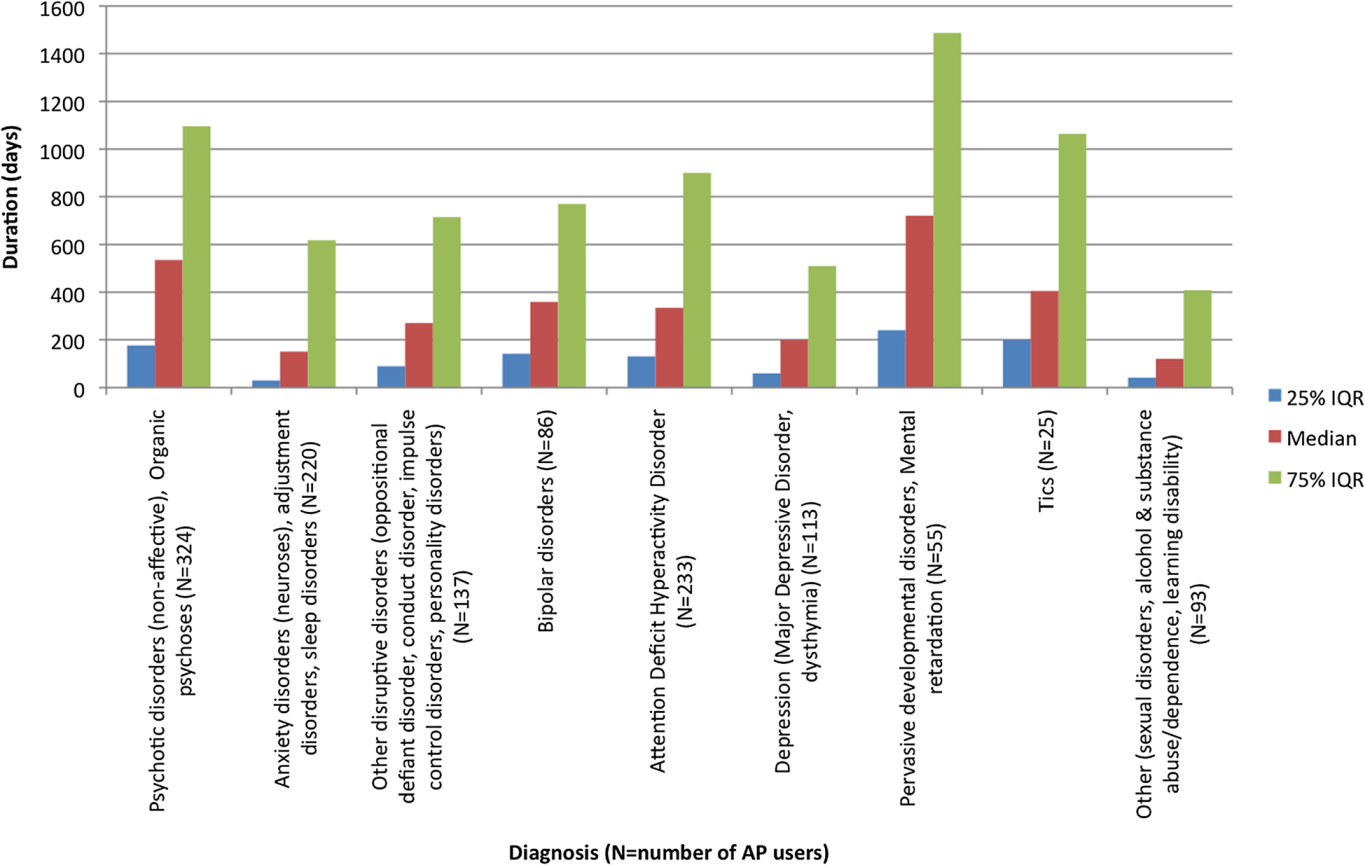
Median and interquartile range of duration of antipsychotic use in days by diagnosis (N ≈ 1287)*. * The number of antipsychotic users is less than 1528 (i.e. the number of people in the cohort at least 365 days) as AP related diagnostic information was not available for all users. The eating disorders category had a cell size of less than five and was eliminated from the graph.

Median duration of use varied based on the diagnosis (Figure 6). Diagnoses with the longest associated use were pervasive development disorders and mental retardation followed by psychotic disorders.

### Co-prescribed psychotropic medications

**Figure 7:**
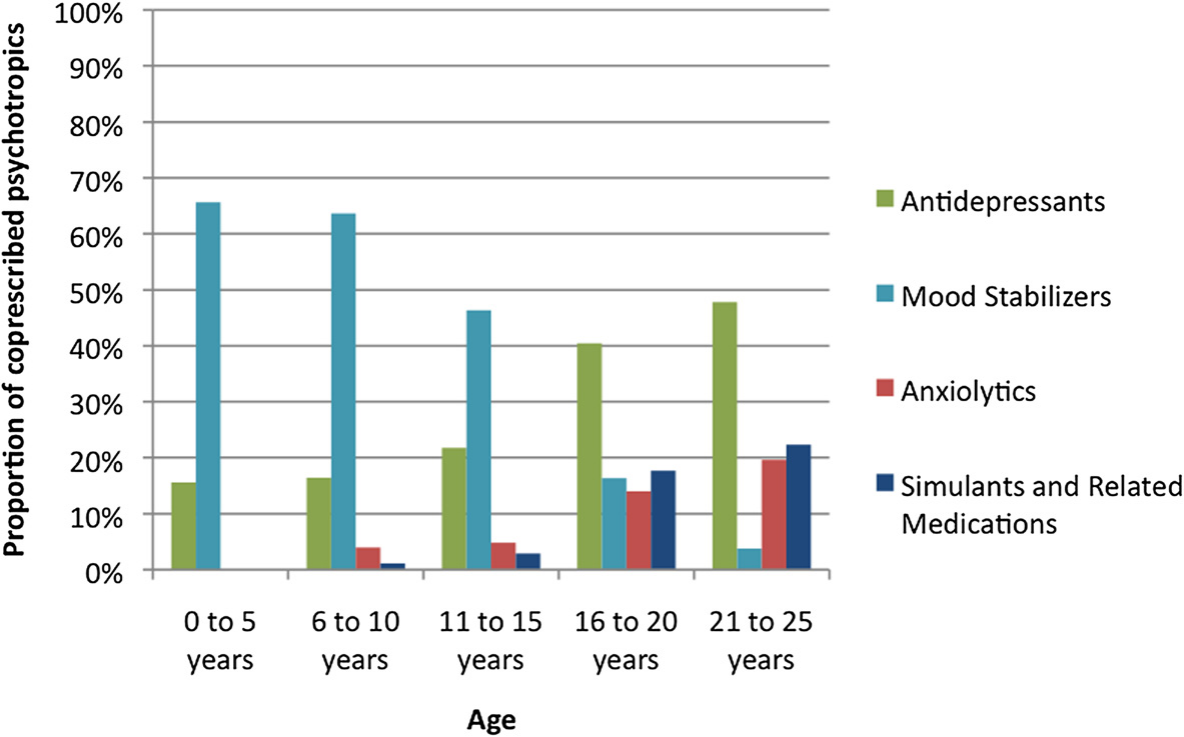
Age stratified use of co-prescribed psychotropics in long-term antipsychotic users (N = 1270) October 1, 2000 (2000Q3) to September 30, 2007 (2007Q2).

Co-prescribing of medications (Figure 7) occurred in long-term AP users and included antidepressants (42%), mood stabilizers (27%), anxiolytics (15%), and ADHD medications (e.g., stimulants atomoxetine, and clonidine) (17%).

### Morbidity and Mortality

Of the 1715 AP users, 796 (46%) were hospitalized at some point during the study period for mental health or behavioral problems. In long-term AP users (N = 1270), there were 64 claimants with a non-mental health diagnosis that did not occur within the previous year of their first incident prescription of APs but did in the two years following. The most commonly recorded diagnosis was amenorrhea (N = 13). Other diagnoses included dyslipidemia and related conditions (N = 10), hypertension (N = 9), diabetes (N = 9), and obesity (N = 8). Other diagnoses include ischemic heart disease, other forms of heart disease, cerebrovascular disease, hyperglycemia NOS, secondary parkinsonism (dystonia), sexual activity problems, and galactorrhea.

**Figure 8:**
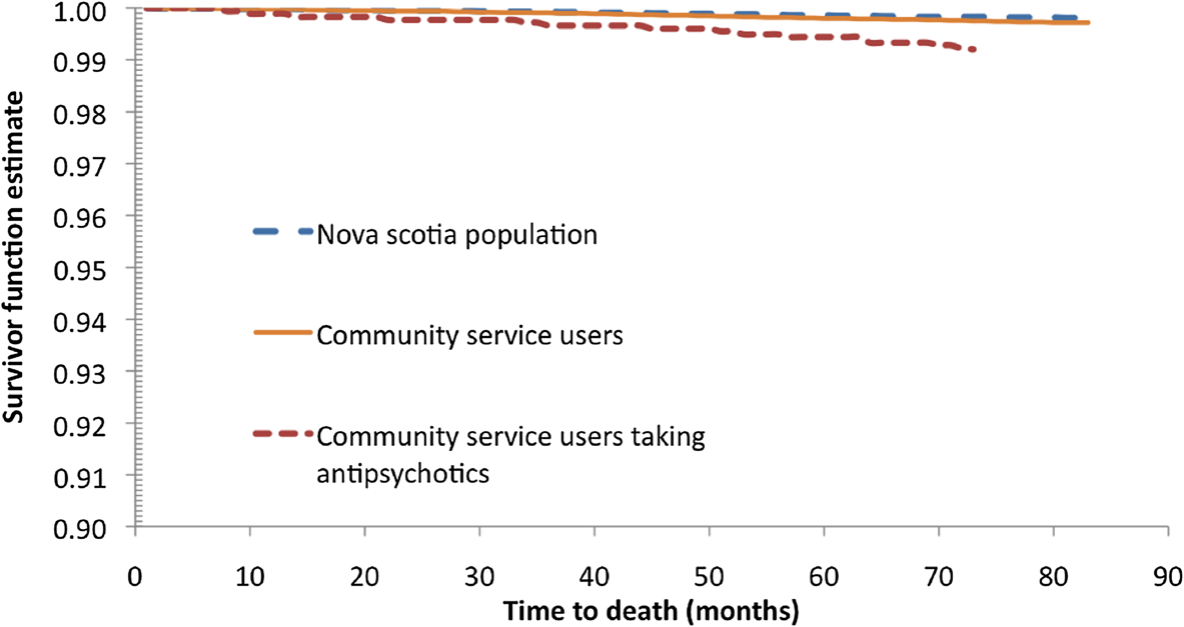
Fitted survival curve for AP recipients, community services recipients and the general population of Nova Scotia, October 1, 2000 (2000Q3) to September 30, 2007 (2007Q2).

There were 16 deaths among the 1715 AP users, 126 deaths in the remainder of the cohort of community services recipients (N = 42,173), and 792 deaths in the rest of the Nova Scotia population aged 0 to 25 (N = 387,202). The age and sex adjusted hazard ratios for death with AP users was 1.70 (95% CI 0.99, 2.90; X^2^ = 3.718 df = 1, p =0.054) compared to the community services population and 3.66 (95% CI 2.23, 6.01; X^2^ = 26.4 df = 1, p < 0.0001) compared to the general population of 0 to 25 year olds. Figure 8 shows the fitted survival curve for AP recipients, community services recipients and the general population of Nova Scotia. For the 16 deaths that occurred in AP recipients, causes of death were related to diabetes, cerebrovascular events, misuse of drugs, and intentional self-harm, among others.

### Costs

The total paid by the publically funded Phamacare program for AP medications rose over time. For example, spending in 2001 was $346,398 and in 2006 was $709,702. The amount paid per AP user by Pharmacare (minus copay) was $879.18 (N = 394 AP users) and $1049.85 (N = 676 AP users), respectively, for those same years.

## Discussion

Although some improvements in building knowledge and capacity in child and youth mental healthcare have been made in the face of formal policies (e.g., the US Best Pharmaceuticals for Children Act and Pediatric Research Equity Act), and frameworks [27], many aspects (e.g., appropriate starting and target dose, duration, etc.,) of pharmacotherapy in children and adolescents are relatively disadvantaged by a lack of reliable, empirically derived data [28]. This is particularly concerning given the disparities that can occur in health services among various subgroups (e.g., low income, visible minority, mental illness) within child and adolescent populations.

Antipsychotics have not been substantially or systematically evaluated prospectively in children and adolescents, but their diffusion of use continues to increase in this population. The prevalent use of SGAs in younger age strata in our study is similar to others [2,14,29]. The causes are likely to be multi-factorial and include, but are not limited to, changes in societal attitudes about medication use, changes in health care system delivery in which resources serving as alternatives to medications are not available, changes in service delivery such that more severe cases of mental problems are managed in the community, putative effectiveness of these compounds, prescriber expectations of therapeutic results, and marketing pressures.

Young people with mental illness may experience inequalities in health service access and delivery and experience suboptimal treatment and health-related outcomes [25,30-41]. Children and adolescents similar to those in our database could experience increased burden of illness related to poorer health as they age due to these inequalities in care. These considerations must also be placed in context of uncertainties and a general lack of understanding regarding the long-term consequences (e.g., cardiometabolic effects) of AP use in this group. Given the enormous direct and indirect cost implications of managing chronic diseases, the implication of the expanded use of APs and other psychotropics in youth needs to be explored more thoroughly. The direct AP costs in our cohort are similar to the results of others [42-45], but we do not know if these added costs were offset by benefits (e.g., fewer hospitalizations, improved clinical or functional outcomes) or were compounded by trade-offs (e.g., managing cardiometabolic side effects). It is apparent that the profile of adverse consequences of these agents are the impetus for changes in some settings of mental health care service delivery in which specific metabolic monitoring programs are in place for children and adolescents [46,47].

We identified several diagnoses in AP users that are concerning from a chronic disease perspective. In the 1270 long-term users aged 0 to 25 years, 64 had non-mental health diagnoses temporally related to APs including sexual and reproductive, cardiometabolic disorders, and movement disorders. With the limitations of claims data in mind [48], these findings of morbidity that are potentially treatment related are notable. Antipsychotic users in the community services database had a significantly higher mortality than the age matched general population of Nova Scotia and neared significance compared to other community services recipients. It cannot be inferred that the higher mortality rate is associated with AP use due to numerous limitations of our methods. Other Canadian researchers have found that children in welfare systems are at higher risk for suicide and suicide attempts [49]. We do not know from our data what, if any (for example, protective, neutral or negative), impact of AP use had on such behaviors but this is an important issue for further study.

Risperidone was the most commonly prescribed SGA in our cohort. There was a rapid rise in risperidone use towards the final quarter of 2003 and later, which followed a change in formulary status of risperidone. On December 1, 2003, Pharmacare changed the benefit status of both risperidone and quetiapine from medications that required specific criteria for use (i.e., restricted use) to an open benefit. Other researchers have shown significant increases in usage and costs occurring with changes in AP formulary reimbursement strategies [42-45]. By contrast, quetiapine did not show a similar increase with the same change in policy. This agent was relatively new to prescribers and, unlike risperidone, had no US FDA pediatric indications [50]. ADHD, a non-Health Canada approved indication, was the most common diagnosis for the use of risperidone in approximately 31% of AP users based on our diagnostic algorithm. Other research has shown similar trends with SGAs used for ADHD and conduct disorder [14,29]. Most AP use in our study was off-label; all use in youth under 18 was off-label as was use in young adults with diagnoses other than psychoses or bipolar disorder (Figure 5).

Co-prescribing of other psychotropic medications was prevalent in our cohort and rates found by other researchers vary [51]. The rationale for co-prescribing likely depends on a host of factors (e.g., physician preference, diagnosis, etc.). The use of mood stabilizers in the 0 to 15 year old group was more prevalent than expected. The presence of co-prescribing in 0 to 5 year olds requires further detailed characterization especially given the lack of prospective evaluation of psychotropic polypharmacy in younger age groups.

In our study, GPs initiated prescriptions for incident APs most of the time, which is consistent with the findings of some [2,29] but not all researchers including those in Canada [14]. This finding requires further ex-plication of GPs’ roles in the care of children and adolescents with mental illness given our findings related to AP indication, dosing and duration. Because of the limited income criteria for eligibility to be in the cohort, AP users were likely only able to receive care from a psychiatrist if referred by a GP. Accessing psychiatrist services through private or uninsured mechanisms would be unexpected because it would be cost-prohibitive. Whether the GPs in our study initiated these prescriptions in consultation or collaboration with, or based on the recommendation of a psychiatrist or pediatrician, is unknown. Some Canadian data have shown that family physicians can operate autonomously in their care of adolescents with mental health problems [52]. We are also unable to determine the characteristics of the prescribing GPs and whether these physicians work in groups, individually, or have formal collaborative arrangements with specialist mental health professionals. Canadian researchers in Quebec developed typologies for GPs or groups of GPs generally involved in caring for those with chronic or serious mental disorders [53]. Characterizing GP prescribers by typology or some other methods could be useful for enhancing capacity and networks, determining efficient models of care, and developing and targeting education and training programs.

The dosing of APs in our study was highly variable. These findings warrant further investigation to determine appropriateness of these doses given the age, diagnoses, and available evidence. Olanzapine dosing appeared higher than dosing of other SGAs perhaps due to its preferential use for the treatment of psychosis. The median duration of AP use was similar across age groups. This finding is noteworthy, as we could have anticipated that the median duration would be shorter in younger children and increase with increasing age as with the findings of others [14]. One explanation is that longer treatment was associated with chronic conditions such as pervasive developmental disorder or learning disability.

We are currently conducting a qualitative study with data analyses nearing completion (to be reported elsewhere). We will use the information from the lived experiences of youth, parents or caregivers, and prescribers vis a vis APs and the results presented in this work to inform healthcare decision and policy making, future research, and clinical practice.

### Limitations

There are inherent limitations to conducting retrospective studies [48] and we will outline those specific to our databases.

The findings of our study may not generalize to the general population of children and adolescents as our population only included those who were eligible to receive a publically funded drug program. We have information on what prescriptions were filled but do not know if and how they were taken. We were not able to capture any information on medications not reimbursed through the publically funded drug plan.

The study period covered seven years and therefore patterns prior to and following the study are unknown, including the effect of the release of newer SGAs. People receiving community services benefits had changes in their eligibility status throughout the study cohort however, 1326/1715 (77%) had a cumulative period in the database from the 2000/01 to 2006/07 fiscal periods of at least 2 years. Given the age group of the cohort and the population with lived experiences with mental illness, periods of hospitalization, changes in employment, and changes in care-giving arrangements could all contribute to changes in eligibility status. We were not able to capture this information on changes in depth but we do know that 46% of the cohort was hospitalized at least once during the study period in relation to their mental health.

Indications for AP use were inferred based on a diagnostic hierarchy. The cause of increased risk for death in the community services AP users is unknown and likely multifactorial.

## Conclusions

Antipsychotic prescribing was high in a low income cohort of youth. The majority of the prescribing was initiated by GPs and would be considered off-label. Co-prescribing of psychotropics was prevalent. The change in the formulary status of risperidone was associated with an increase in prescribing. This study raises questions about the appropriateness of prescribing given the wide range of diagnoses, doses, duration and combinations of treatments for all ages, especially those less than 5 years old. The cumulative body of research regarding AP use in youth demonstrates that there are gaps in knowledge regarding safe and effective use of these agents. Translation and implementation of knowledge to inform policy and practice towards appropriate use is needed.

## Abbreviations

AP: Antipsychotic; ADHD: Attention Deficit Hyperactivity Disorder; ATC: Anatomical Therapeutic Classification; FGA: First generation antipsychotic; ICD: International Classification of Diseases; SGA: Second generation antipsychotic.

## Competing interests

The authors declare that they have no competing interests.

## Authors’ contributions

ALM conceived the idea for the study and initial design for quantitative and qualitative phases. In collaboration with DMG, CC, SK, and SPK, the quantitative design was refined and in collaboration with JH the qualitative design was further developed. ALM, CC, and DMG participated in all steps of quantitative study procedures, conduction, and data verification, analyses and interpretation. ALM, DMG and CC had full access to all of the aggregated data for the study and were responsible for initial interpretation. JH, SK, and SPK contributed to reviewing and augmenting the interpretation of quantitative findings following the first manuscript draft. All authors have contributed critical review and feedback and approved the final version of the manuscript.

## Authors’ information

ALM is an Associate Professor with the College of Pharmacy and Department of Psychiatry at Dalhousie University. She worked as a clinical pharmacist in mental health with inpatient and outpatient youth and is a team member of the Sun Life Financial Chair in Adolescent Mental Health. DMG is a Professor with Dalhousie University Department of Psychiatry and works clinically as a pharmacist with the Nova Scotia Early Psychosis Program. DMG also volunteers extensively making contributions regarding medication and condition-related knowledge for support groups of those with lived experiences of mental illness. He was a member of the Science Advisory Committee for the Mental Health Commission of Canada and currently Chairs the Awards Committee on Research for the Commission. CC is a Masters prepared (Community Health and Epidemiology) practicing community pharmacist with extensive training and expertise in health related databases. She conducts pharmacoepidemiology and related epidemiology health services research. She has been a member of the Drug Evaluation Alliance of Nova Scotia (DEANS) since 2006. At the time of the research, CC was employed at the Population Health Research Unit. SK is trained in addiction, public health medicine and psychiatry. He is currently practicing in Australia as a Professor and the Director of Health LinQ at the University of Queensland while maintaining an adjunct appointment with Dalhousie University's Departments of Psychiatry, Community Health and Epidemiology. His work as a clinician and scholar inform one another and he has focused on researching mental health services delivery, epidemiology/pharmacoepidemiology, and psychiatric co-morbidity. JH is a Professor in the School of Nursing at Dalhousie University and a Research Scientist with the Department of Psychiatry at the IWK Health Centre. She is also Senior Editor for the Canadian Journal of Community Mental Health. Her research and publications concentrate on marginalized populations (high inequalities, homeless) of children and youth with a focus on mental health issues. She is closely linked to her advocacy work at the provincial and national levels with professional regulatory boards, the Canadian Mental Health Association, Laing House, and a number of other community organizations. SPK holds the Sun Life Financial Chair in Adolescent Mental Health and directs the World Health Organization Collaborating Center in Mental Health at Dalhousie University. He holds numerous awards and honors locally, nationally and internationally for his work including: Best Doctor in Canada; Dr. John Savage Memorial Award for outstanding humanitarian contributions to global health; Canadian College of Neuropsychopharmacolgy Gold Medal; membership in the Canadian Academy of Health Sciences; and the Lifetime Achievement Award of the Canadian Psychiatric Research Foundation. He has been appointed a Fellow of the Canadian Psychiatric Association. He is a member of the Institute of Neuroscience, Mental Health and Addictions of the Canadian Institutes for Health Research; Interhealth Canada; Mental Health Commission of Canada (Child and Youth Advisory Committee); the Canadian Society for International Health; and the Canadian Coalition for Global Health Research. He is leading the development of a Canadian child and youth mental health framework: Evergreen. His current focus is on knowledge translation pertaining to youth mental health as it is applied in schools and primary care.

## Pre-publication history

The pre-publication history for this paper can be accessed here:

http://www.biomedcentral.com/1471-244X/13/198/prepub
